# Zinc and metformin co-functionalized polyetheretherketone: A novel dental implant material tailored for the elderly

**DOI:** 10.1016/j.jare.2025.08.056

**Published:** 2025-08-27

**Authors:** Zhengwei Liu, Enze Zhao, Yansong Wang, Hanwei Huang, Yuxun Wu, Yichong He, Shuting Bai, Suwen Wang, Shirou Fan, Shuaishuai Cao, Bin Tang

**Affiliations:** a Department of Orthopedics, Northern Jiangsu People’s Hospital, Clinical Teaching Hospital of Medical School, Nanjing University, Yangzhou, Jiangsu, PR China; b Department of Biomedical Engineering, Southern University of Science and Technology, Shenzhen, Guangdong, PR China; c Department of Stomatology, Shenzhen Hospital (Futian) of Guangzhou University of Chinese Medicine, Shenzhen, Guangdong, PR China; d ShenZhen College of International Education, Shenzhen, Guangdong, PR China; e The Hong Kong University of Science and Technology (Guangzhou), Guangzhou, Guangdong, PR China; f Department of Stomatology, Shenzhen University General Hospital, Shenzhen University, Shenzhen, Guangdong, PR China

**Keywords:** Dental implant, Polyetheretherketone, Osseointegration, Osteoblast senescence, Surface modification

## Abstract

**Introduction:**

The rising demand for dental implants among the elderly population is challenged by age-related impairments in osteogenic regeneration, such as Wnt/β-catenin signaling decline, ROS-mediated cellular senescence (compromising osteoblast differentiation), and elevated pro-inflammatory cytokines (e.g. IL-6, TNF-α). Senescent osteoblasts establishes a pathological triad: (1) cell-cycle arrest-mediated osteogenic impairment, (2) SASP-fueled chronic inflammation, and (3) immune-bone uncoupling—collectively driving alveolar bone deterioration in the elderly. Polyetheretherketone (PEEK), with its elastic modulus similar to that of alveolar bone, emerges as a promising implant material. However, its bioinert nature limits its osseointegration capabilities.

**Objectives:**

This study aimed to develop a functionalized PEEK derivative, ZnMet@PEEK, by integrating zinc (Zn) and metformin (Met) into PEEK. to enhance osseointegration with alveolar bone in elderly patients.

**Methods:**

In this study, we combined the anti-inflammatory and osteogenic properties of Zn with the anti-senescence effects of Met using dopamine-assisted physical adhesion to prepare ZnMet@PEEK, aiming to enhance PEEK’s osseointegration with alveolar bone in elderly patients. We examined the biocompatibility and osteoblast differentiation-inducing ability of ZnMet@PEEK *in vitro* by CCK8, live/dead cell staining, RT-qPCR, immunofluorescence staining and Enzyme-Linked Immunosorbent Assay for osteogenesis-related proteins. Further, we constructed an animal model and detected the bone regeneration ability of ZnMet@PEEK *in vivo* by micro-CT and histological analysis.

**Results:**

Material characterization confirmed the incorporation of ZnMet onto PEEK. *in vitro* and *in vivo* evaluations demonstrated that ZnMet@PEEK exhibited excellent biocompatibility, significantly inhibited inflammation, promoted bone formation, and inhibited cellular senescence, ultimately resulting in enhanced osseointegration in aged rats.

**Conclusions:**

In this study, we successfully developed a new functionalized PEEK derivative, ZnMet@PEEK, with tailored bioactivities including anti-inflammatory, osteogenic, and anti-senescence properties, as a dental implant for the elderly population. Therefore, we believe that ZnMet@PEEK, specifically tailored for elderly patients, is a promising dental implant material offering a novel solution to the multifaceted challenges of osseointegration.

## Highlights

• ZnMet@PEEK enhances PEEK implants for elderly patients via Zn and metformin loading.
• The surface exhibits high wettability and sustained Zn/Met release.
• ZnMet@PEEK boosts osteogenesis by upregulating RUNX-2, Col-1, and BMP-2.
• It reduces IL-1β, IL-6, and P21, mitigating inflammation and senescence.
• In vivo, ZnMet@PEEK improves osseointegration and shows excellent biosafety.

## Graphical abstract

**Figure:**
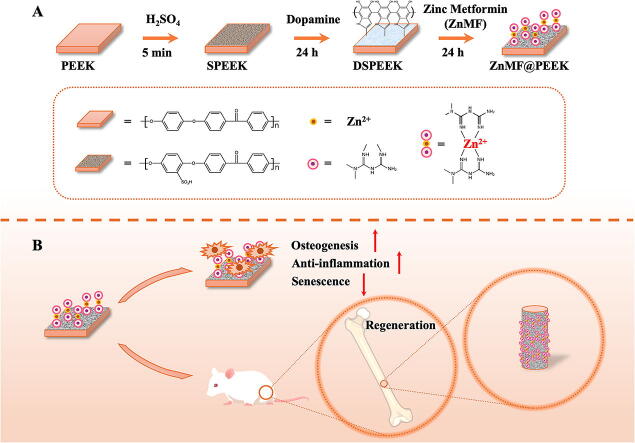

## Introduction

Imagine an 80-year-old patient, Mrs. Chen, presenting with repeated dental implant failures due to bone resorption and chronic inflammation, a distressing reality for many elderly individuals seeking to restore their quality of life [1]. Elderly patients, representing a growing demographic in dentistry, frequently experience tooth loss and compromised bone regeneration, leading to high rates of dental implant failure [2]. While titanium implants remain the gold standard due to biocompatibility and strength [3], their high elastic modulus (∼110 GPa) causes significant stress shielding against aging bone (∼1.8 GPa), promoting bone resorption and implant failure [4,5]. Age-related impairments critically hinder osseointegration: chronic inflammation (elevated IL-1β, IL-6, TNF-α activating NF-κB) promotes bone resorption and inhibits osteoblast differentiation [[6], [7], [8]]; oxidative stress (excess ROS) suppresses osteogenic regulators (RUNX-2, osterix) [9,10]; and cellular senescence (upregulated p16/p21, SASP) amplifies inflammation and degrades the extracellular matrix [11]. Furthermore, diminished BMP and Wnt/β-catenin signaling reduces expression of osteogenic genes (e.g., Col-1, ALP). These complex molecular mechanisms collectively result in prolonged recovery, increased failure rates, and reduced patient satisfaction, underscoring the urgent need for innovative implant materials specifically designed to overcome the unique physiological challenges of the elderly.

Polyetheretherketone (PEEK) presents a promising alternative to titanium implants due to its favorable mechanical properties, notably an elastic modulus (3–4 GPa) closely matching alveolar bone (1–2 GPa), which minimizes stress shielding—a critical advantage for the elderly [12,13]. However, PEEK's inherent bioinertness limits osseointegration, particularly in aging tissues with impaired regenerative capacity [14]. While surface modifications like hydroxyapatite coatings enhance bioactivity, they often fail to address age-specific challenges such as chronic inflammation and cellular senescence [15,16]. Consequently, functionalizing PEEK with bioactive agents capable of simultaneously promoting osteogenesis and mitigating age-related pathological processes is essential for developing truly effective elderly-tailored dental implants [[17], [18], [19]]. Zinc (Zn), an essential trace element, exhibits potent therapeutic effects for bone regeneration. Zn ions suppress inflammation-induced bone loss by modulating NF-κB signalling [20]. Crucially, Zn enhances osteoblast differentiation and mineralization via activation of the Wnt/β-catenin pathway. Recent evidence suggests Zn may further potentiate Wnt signaling by targeting mechanisms like the miR-665/SOST axis, which alleviates osteoporosis by inhibiting the Wnt antagonist SOST [[21], [22], [23], [24]]. These dual anti-inflammatory and osteoinductive properties make Zn an ideal bioactive component for functionalizing PEEK dental implants, particularly to address the compromised bone healing capacity in elderly patients. Metformin (Met), a repurposed anti-diabetic drug, exhibits potent anti-senescence and osteogenic properties relevant to dental implants [25,26]. Met activates AMPK, improving mitochondrial function and reducing oxidative stress, while inhibiting mTOR to suppress senescent cell accumulation and the detrimental SASP [27,28]. Crucially, Met enhances bone formation by upregulating BMP signaling and RUNX-2 expression in mesenchymal stem cells (MSCs), potentially extending osteoblast longevity—a key need in the elderly [[29], [30], [31]]. The synergy between Zn and Met arises from their complementary actions: Zn creates a pro-osteogenic microenvironment through anti-inflammatory and Wnt-mediated effects, while Met directly combats senescence and boosts osteoblast function [[32], [33], [34], [35]]. This combined approach, demonstrated to improve bone regeneration in aged models by simultaneously targeting inflammation, senescence, and osteogenesis, offers a promising strategy to enhance implant success in elderly patients with compromised healing.

**Fig. 1:**
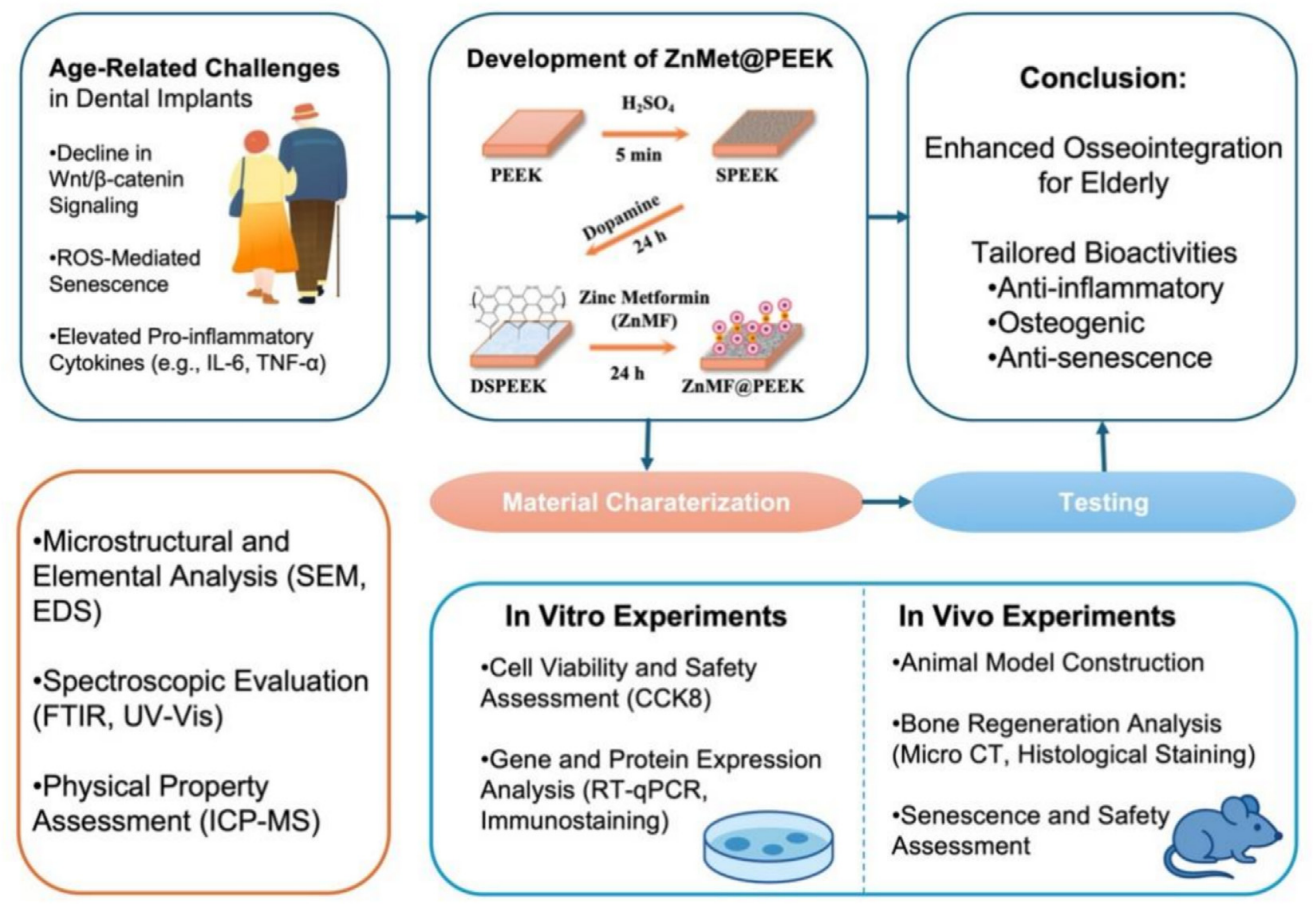
Schematic representation of the research workflow for developing and evaluating ZnMet@PEEK as a dental implant material for elderly patients. The strategy integrates material synthesis via sulfonation and ZnMet functionalization, comprehensive physicochemical characterization, and sequential *in vitro* and *in vivo* assessments. This interdisciplinary approach targets age-related impairments in bone healing by enhancing anti-inflammatory, osteogenic, and anti-senescent responses.

To address the multifaceted challenges of dental implant therapy in the elderly, a functionalized PEEK derivative, ZnMet@PEEK, has been developed by incorporating Zn and Met onto the PEEK surface using a dopamine-assisted physical adhesion method. Fig. 1 outlines the overall strategy of the paper and how the research is conducted. This surface modification strategy seeks to enhance PEEK's bioactivity by leveraging the synergistic effects of Zn and Met. A series of physicochemical characterizations have been conducted to confirm the successful preparation of ZnMet@PEEK. Additionally, comprehensive *in vitro* and *in vivo* experiments have been performed to systematically evaluate its biocompatibility and bioactivity. The results indicate that ZnMet@PEEK is a promising candidate for dental implant materials in the elderly population, addressing issues related to diminished osteogenic potential, persistent inflammation, and osteoblast senescence.

## Experimental details

### Materials and equipment

Met was purchased from Bio-Year Technology Co., Ltd. (China), PEEK was purchased from Victrex Technology Company (UK), and dopamine, NaOH, ZnCl_2_, and cation exchange resin were all purchased from Sigma-Aldrich Company (China). All reactants were used as received. Deionized water (> 18.2 MΩ·cm) was used when water was involved. The biochemical reagents and experimental equipment involved in this research are shown in Table S1 and Table S2.

### Preparation of ZnMet@PEEK

Zinc metformin (ZnMet) was prepared before surface modification of PEEK using the following procedure. Initially, a saturated aqueous solution of Met was combined with an equal volume of a 2 M aqueous ZnCl_2_ solution. The resulting mixture was then stirred with NaOH to adjust the pH to a range of 9 to 11, and this was maintained for 1 h to ensure a complete reaction. Following this, absolute ethanol was added to the reaction mixture, which was then allowed to stand for 24 h to facilitate the full precipitation of the composite powder. The precipitated composite powder was subsequently filtered using a vacuum suction apparatus. After filtration, the powder was stirred and washed with ethanol aqueous solutions of 80 %, 60 %, and 40 % concentrations, respectively. To guarantee the complete removal of any residual ZnCl_2_, the washing process was repeated three times to obtain ZnMet with high purity. Before the introduction of ZnMet, PEEK samples were washed with acetone, absolute ethanol, and deionized water under ultrasound for 10 min, and then dried in an incubator at 60 °C for 24 h. Afterwards, the PEEK samples were sulfonated by immersing them in 98 % concentrated sulfuric acid and ultrasonically vibrating them at 25 °C for 5 min. After sulfonation, the PEEK samples were cleaned under ultrasonic conditions with a ratio of 5 ml of deionized water for 10 min twice to remove residuals. After cleaning, they were dried in a constant temperature oven at 60 °C for 24 h. The dried sulfonated PEEK sample was named SPEEK. An aqueous dopamine solution was prepared by dissolving dopamine in Tris buffer at a concentration of 2 mg/mL and pH 8.5. The SPEEKs were soaked in the aqueous dopamine solution for 24 h to introduce dopamine onto the SPEEK surface and then rinsed with deionized water to remove any unattached residues. The SPEEK after the introduction of dopamine was thereafter named DSPEEK. A 200 μL volume of ZnMet solution was administered onto the surface of DSPEEK and allowed to remain at room temperature for 24 h to ensure that ZnMet was fully bound to DSPEEK. This process resulted in the formation of PEEK with incorporated ZnMet, which we designated as ZnMet@PEEK. Subsequently, these samples underwent freeze-drying for a period of 48 h. To investigate the effects of varying ZnMet concentrations, we prepared a series of ZnMet@PEEK samples using ZnMet solutions with concentrations of 100, 300, 500, 1000, and 1500 μg/mL.

### Materials characterizations

The microstructure of the samples was observed using a scanning electron microscope (JSM-7200F, JEOL company, Japan). The surface microstructure of PEEK samples and prepared samples was observed by scanning electron microscopy (SEM, Nova NanoSem450), the elemental composition and element distribution of the surface of different samples were measured by energy dispersive spectrometer (EDS) (Nova NanoSem450 FEI, USA). Since the samples are non-conductive, prior to SEM observation, gold spraying was performed to enhance conductivity and optimize image clarity. In addition to EDS, attenuated total reflection Fourier transform infrared spectroscopy (ATR-FTIR) (VERTEX 70v, German) and Ultraviolet–visible spectroscopy (UV–Vis) were employed to further investigate the chemical structure (UV-2600 SHIMADZU, Japan).

The Zn ion release behavior of the samples was investigated using inductively coupled plasma-optical emission spectrometry (ICP-MS) (Agilent 7900 ICP-MS, USA). Samples were soaked in physiological saline at 37 °C to simulate the *in vivo* environment. Aliquots were collected at pre-designed time points over a 72-hour period, and the concentration of Zn ions in each aliquot was measured respectively.

The surface hydrophilicity of the samples was quantitatively assessed using a contact angle system (VCA OPTIMA, AST) In the contact angle measurement, three samples from each group were used, with measurements taken at five randomly selected points on each sample to improve the reliability of the results.

### Cell culture

Mouse embryonic osteoblasts MC3T3-E1 cells were used in this study. The culture medium used was α-MEM containing 1 % penicillin–streptomycin and 10 % FBS, and the cells were cultured in a cell incubator under standard culture conditions (37 °C, 5 % CO_2_). The culture medium was replaced every 2 days to support healthy cell growth and differentiation potential. After 5 days, cells were passaged to avoid contact inhibit.

### Cell viability assessment

In this study, the CCK8 method was used to evaluate the cell viability of samples and to determine the optimal drug concentration. During the experiment, MC3T3-E1 cells were first evenly seeded in a 96-well plate at a density of 1 × 10^4^ cells/well, and an appropriate amount of culture medium was added. Incubate under constant temperature culture conditions of 37 °C and 5 % CO_2_. After one day of cell attachment growth, the original medium was replaced with culture medium containing different sample concentrations, including control group with PEEK (NC group), DSPEEK group, and ZnMet@PEEK synthesized at 100, 300, 500, 1000, and 1500 μg/ml. Each sample was immersed in 15 ml of complete medium and shaken in a shaker at 37°C for 24 h to get sample effusion. Then, cell viability was detected on days 1, 3, and 5 using the CCK8 kit. The dead/live cell staining by Calcein-AM/PI Double Straining Kit (Beyotime, China), and an inverted fluorescence microscope was employed to observe stained cells.

### Examination of gene expression

Real-Time Quantitative Reverse Transcription PCR (RT-qPCR) was employed to assess the expression levels of genes before and after implant modification. MC3T3-E1 cells were seeded onto sterilized DSPEEK placed in a 6-well plate at a density of 1 × 10^5^ cells per well and cultured for 7 days. MC3T3-E1 cells' RNA was extracted by applying RNAiso plus according to the instructions. We determined the concentration of total RNA extracted from samples in each group using Nanodro 2000 and synthesized cDNA. The synthesized cDNAs were amplified using an RT-qPCR system (Applied Biosystems). Expression levels of genes (Col-Ⅰ, Runx2, ALP, IL-6, IL-1β, BMP-2, OCN, OPN, TNF-α, p16, p21) were normalized using GAPDH. The primers utilized in the study are listed in Table 1.

**Table 1:** The sequence of the primer for RT-PCR.

Gene name	Forward primer (5′-3′)	Reverse primer (5′-3′)
GAPDH	AACGACCCCTTCATTGAC	TCCACGACATACTCAGCAC
RUNX-2	ATGAGAGTAGGTGTCCCGCC	GTGGAGTGGATGGATGGGGA
IL-6	TCCATCCAGTTGCCTTCT	TAAGCCTCCGACTTGTGA
IL-1β	GAAATGCCACCTTTTGACAGTG	TGGATGCTCTCATCAGGACAG
Col-1	ACGCCATCAAGGTCTACTGC	ACTCGAACGGGAATCCATCG
ALP	CGAGAGTGAACCATGCCACC	AGCTGGTAGGCGATGTCCTT
BMP-2	ACTCGAAATTCCCCGTGACC	CCACTTCCACCACGAATCCA
OCN	CAAGTCCCACACAGCAGCTT	AAAGCCGAGCTGCCAGAGTT
OPN	ATCTCCTTGCGCCACAGAATGC	CGTCAGATTCATCCGAGTCCACAG
TNF-α	CTCTTCTGTCTACTGAACTTC	CTCCTGGTATGAGATAGCAA
p16	TCAAGACATCGTGCGATATTTG	TTAGCTCTGCTCTTGGGATTG
p21	ATGTCCAATCCTGGTGATGTC	GAAGTCAAAGTTCCACCGTTC

### Protein expression evaluation

Put it simply, MC3T3-E1 cells were incubated separately with Rabbit Polyclonal Antibody (ALP, OCN, RUNX-2, BMP-2, p16) (1:100 dilutions) overnight at 4°C. Subsequently, they were exposed to FITC/Cy5-Labeled secondary antibody individually for 60 min. Finally, the nucleus was stained with DAPI staining solution and treated with anti-fluorescence quenching. Pictures were taken with a LEICA fluorescence microscope.

For the ELISA test, the standard product series, according to the ELISA kit instructions, were added to the 96-well plate. Then, biotinylated antibodies were added and incubated. Streptavidin labeling solution was added after washing, and TMB substrate solution was added after rewashing and incubated for 20 min away from light. Finally, a termination solution was added to terminate the reaction. To get the results, the absorbance (OD value) of each hole was measured by the microplate reader at 450 nm wavelength, and the concentration of target cytokines in each sample was calculated according to the standard curve.

### Animal model

To investigate dental implant integration and healing performance, 18 aged Sprague Dawley (SD) male rats, 92 weeks old and weighing 350 g, were utilized to create a femoral defect model mimicking the osteointegration of dental implants. The animals were randomly divided into six groups: Control (4 W, 8 W), PEEK (4 W, 8 W), and ZnMet@PEEK (4 W, 8 W). Following anesthesia with isoflurane inhalation, a uniformly sized and positioned bone defect (ø2 mm × 3 mm × 3 mm) was surgically created on the right femur of each rat to simulate the dental implant environment. The defect was then cleansed with physiological saline and filled with PEEK samples matching its dimensions. Post-implantation, the surgical incision was sutured and disinfected with iodophor, and all animals received intraperitoneal injections of penicillin (100,000 units kg^−1^) to prevent infection. The animal experiments were ethically approved by the Shenzhen Institute for Drug Control with the accreditation number: 20220829. Samples were fixed in 4 % PFA for later micro-CT test. Subsequently, the specimens underwent decalcification using a 12 % solution of Ethylene Diamine Tetraacetate (EDTA) and were subsequently dehydrated in ethanol for histological staining, including H&E staining, Masson staining, immunofluorescence staining for Runx2, immunohistochemical staining for P53 and P21, and β-galactosidase staining.

### Statistical analysis

All the images analysis were processed and analyzed with Image J software. The statistically significant difference in experiment data was analyzed with Student’s T-test. All values involved in the study were denoted as mean ± standard deviation (SD). *p* < 0.05 was regarded as the appearance of a significant difference in the data.

## Results

### Materials characterizations of various PEEK samples

**Fig. 2:**
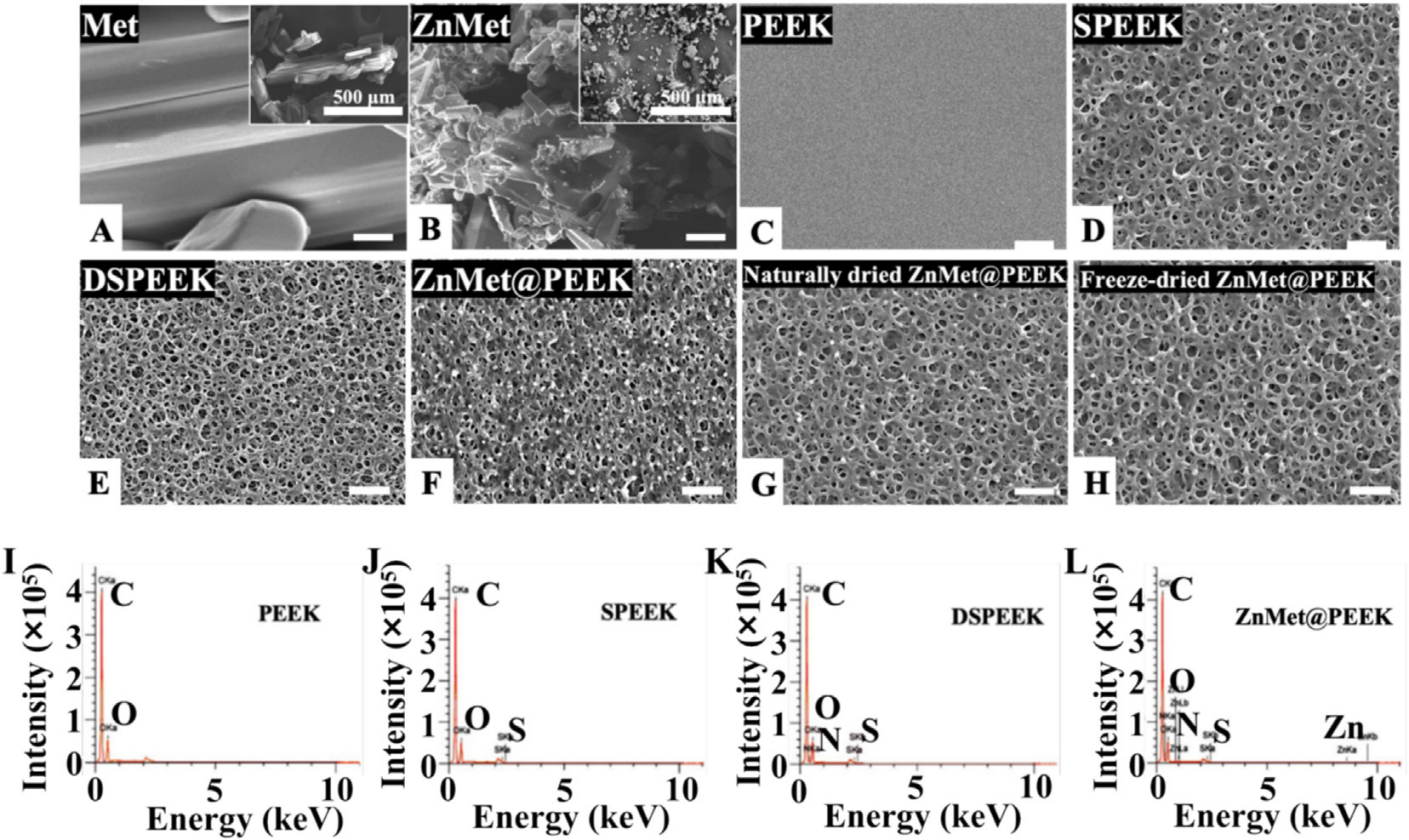
Surface morphology evolution and elemental characterization confirm successful ZnMet functionalization of PEEK. (A–B) SEM images show pristine Met with prismatic crystals and ZnMet with irregular aggregates, confirming successful complexation. (C–F) Surface topographies reveal that sulfonation transforms smooth PEEK (C) into a porous structure (D, SPEEK), which remains after dopamine treatment (E, DSPEEK) and ZnMet loading (F, ZnMet@PEEK). (G–H) Both naturally dried and freeze-dried ZnMet@PEEK retain the interconnected porous morphology, indicating structural stability regardless of drying method. (I–L) EDS spectra illustrate compositional differences across materials: PEEK (I) and SPEEK (J) show only C and O peaks; DSPEEK (K) adds N and S from dopamine and sulfonation; ZnMet@PEEK (L) uniquely exhibits Zn, confirming successful ZnMet incorporation.

Fig. 2 systematically characterizes the surface morphology and elemental composition of various samples. SEM observations reveal distinct structural evolution: pristine Met displays regular prismatic crystals (Fig. 2A), while zinc-modified ZnMet undergoes significant structural disordering into granular aggregates (Fig. 2B). While pure PEEK maintains a smooth surface (Fig. 2C), sulfonation induces structural reorganization into a three-dimensional interconnected porous network (SPEEK, Fig. 2D) with pore sizes spanning 0.5–5 μm. Subsequent processing yields DSPEEK (Fig. 2E) and ZnMet@PEEK composites (Fig. 2F), both retaining comparable microstructural features. ZnMet@PEEK demonstrates slightly reduced pore dimensions (0.5–2 μm) with uniformly dispersed granular particulates adhering to its surface. Comparative analysis of drying methods, e.g. natural drying vs. freeze-drying, confirms minimal morphological differences in ZnMet@PEEK, as both treatments preserve the microporous architecture (Fig. 2G and 2H). EDS result provides critical compositional evidence: zinc signatures remain undetectable in PEEK derivatives (Fig. 2I–K), whereas ZnMet@PEEK exhibits distinct zinc elemental distribution (Fig. 2L), suggesting the successful surface immobilization of ZnMet on the DSPEEK matrix.

**Fig. 3:**
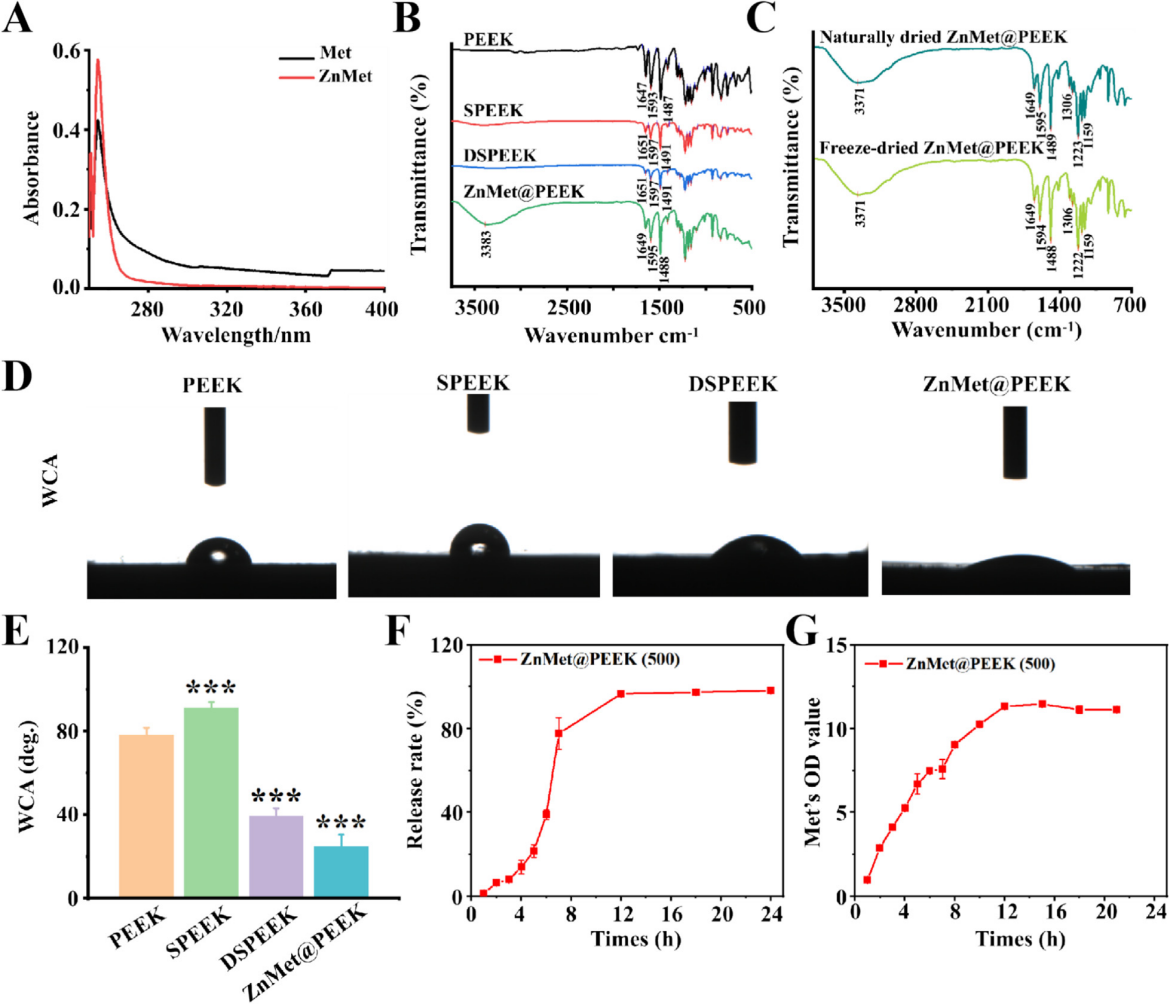
Physicochemical evidence of ZnMet incorporation, surface wettability improvement, and controlled release behavior of ZnMet@PEEK. (A) UV–Vis spectra show enhanced absorbance intensity in ZnMet compared to Met, indicating successful complexation. (B) FTIR analysis reveals the appearance of a new N–H stretching peak (3364 cm^−1^) in ZnMet@PEEK, confirming ZnMet immobilization; sulfonation (SPEEK) introduces SO_3_-related peaks, while dopamine treatment (DSPEEK) preserves surface chemistry. (C) Comparable FTIR spectra of naturally dried and freeze-dried ZnMet@PEEK indicate structural stability across drying methods. (D–E) Contact angle images and quantitative data show significantly enhanced hydrophilicity in ZnMet@PEEK, promoting better fluid–tissue interaction. (F–G) ZnMet@PEEK displays rapid initial release of Zn^2+^ and metformin, reaching a plateau by 12 h, demonstrating effective surface loading and release kinetics suitable for early-stage osteoimmune modulation.

The UV–Vis absorption spectra presented in Fig. 3A depict the optical properties of Met and ZnMet. Both Met and ZnMet exhibit distinct absorption peaks centered around 255 nm, indicative of electronic transitions within their respective molecular structures. Notably, the absorption peak of ZnMet is observed to be more pronounced and exhibits a higher absorbance intensity compared to that of Met. This observation suggests an enhancement in the UV absorption properties of Met upon zinc complexation, potentially attributed to the altered electronic configuration and/or increased conjugation within the ZnMet. Fig. 3B compares the FTIR spectra of PEEK and its derivatives. PEEK exhibits prominent peaks at 1647, 1593, and 1487 cm^−1^, attributed to the aromatic backbone vibrations [1,2]. SPEEK shares similar spectral features with PEEK but exhibits an additional peak at 1051 cm^−1^, indicative of the successful sulfonation process and the presence of sulfonic acid groups [3]. DSPEEK retains the spectral profile of SPEEK, suggesting minimal changes to the bulk structure upon dopamine modification. Notably, ZnMet@PEEK displays a novel peak at 3364 cm^−1^, which is due to the N–H symmetric stretching vibrations and should be the results of the introduction of ZnMet. The EDS data shown in Fig. 2 reveal the presence of zinc, while the FTIR spectra show novel peaks attributable to ZnMet. This combined evidence supports the effective integration of ZnMet into the PEEK, and yield ZnMet@PEEK. We also tested the FTIR spectra of the naturally dried ZnMet@PEEK and freeze-drying ZnMet@PEEK (Fig. 3C). Fig. 3C shows that the FTIR spectra of the naturally dried ZnMet@PEEK and freeze-drying ZnMet@PEEK are similar, which means that different drying treatments do not affect the way ZnMet binds to PEEK. Fig. 3D presents optical photographs showing the typical optical images during the contact angle test for different PEEK samples and Fig. 3E is quantitative results of the contact angle data. The contact angle for pure PEEK is smaller than that of SPEEK. However, when compared to DSPEEK, PEEK displays a larger contact angle, suggesting relatively poorer wettability [4]. Remarkably, ZnMet@PEEK demonstrates the smallest contact angle in the series, indicating the most enhanced wettability among all the tested samples. ZnMet@PEEK's high hydrophilicity, as evidenced by its smallest contact angle, is likely to enhance its bioactivity by promoting better interaction with biological fluids and cells. The results presented in Fig. 3F show the cumulative Zn ion release profile of the ZnMet@PEEK sample over a 24-hour period. It was observed that Zn ions were rapidly released within the first 8 h, followed by a gradual slowing down of the release rate, reaching a plateau phase after approximately 12 h. In addition, we examined the release process of metformin. As shown in Fig. 3G, the release of metformin increased with time, peaked and stabilized by 12 h. This means that the release of metformin on ZnMet@PEEK was completed after 12 h, which is similar to the release process of zinc ions.

### *In vitro* assessment the bioactivities of various PEEK samples

**Fig. 4:**
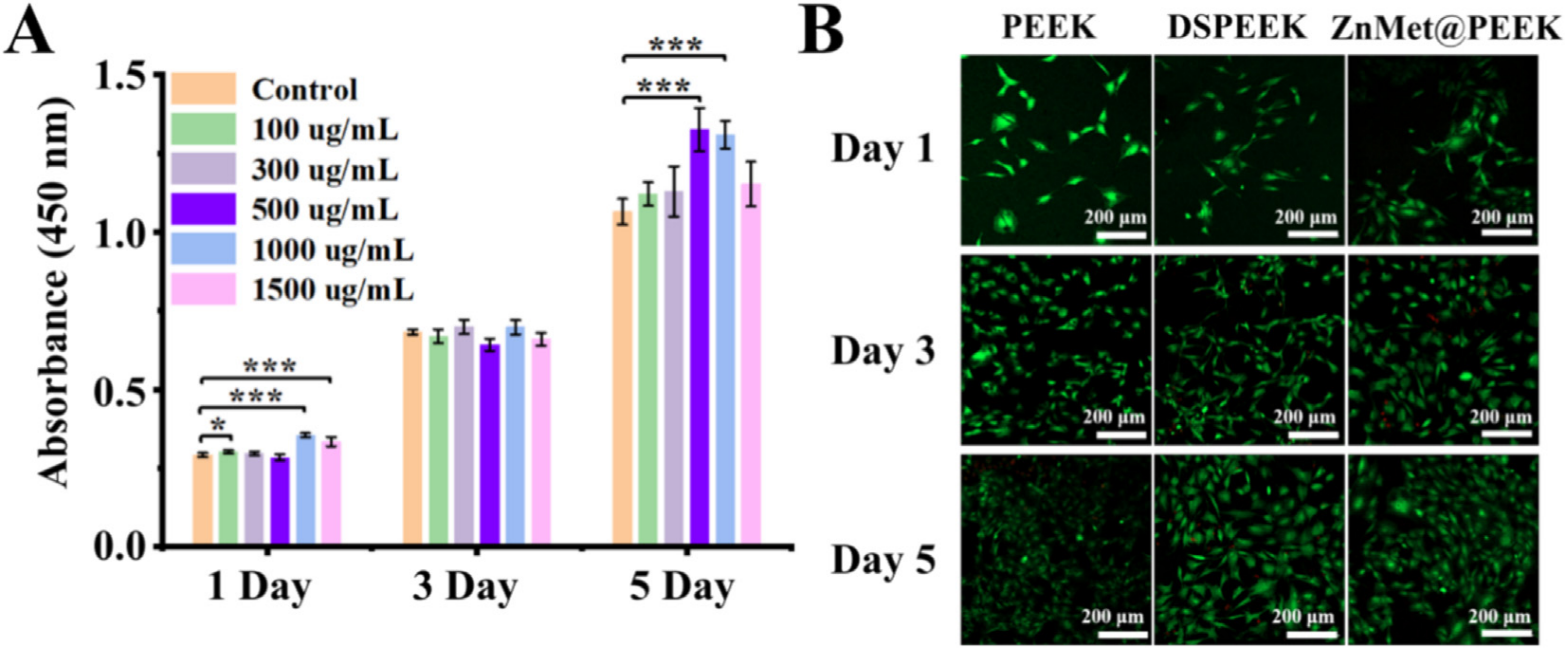
ZnMet@PEEK promotes MC3T3-E1 cell viability and exhibits no cytotoxicity across concentrations. (A) CCK-8 assay shows dose- and time-dependent enhancement of cell viability in ZnMet@PEEK-treated groups; 500 μg/mL group displays the highest absorbance on Day 5, indicating optimal bioactivity without cytotoxic effects. (B) Live/dead staining of MC3T3-E1 cells cultured on PEEK, DSPEEK, and ZnMet@PEEK (500 μg/mL) at Day 1, 3, and 5 reveals time-dependent proliferation with negligible dead cells, confirming favorable biocompatibility of ZnMet@PEEK.

The cell viability and potential toxicity of various PEEK samples were investigated using MC3T3-E1 cells, with DSPEEK serving as the control group, as shown in Fig. 4. Fig. 4A presents the cell viability data of ZnMet@PEEK samples prepared with different ZnMet concentrations. Consistent with the control group, the absorbance values, which reflect the count of living cell number, for all ZnMet@PEEK samples significantly increased over time, preliminarily indicating the safety of ZnMet@PEEK. Regarding the absorbance values across groups, no statistically significant differences were observed on days 1 and 3. However, on day 5, the absorbance values for the 500 μg/ml and 1000 μg/ml groups were significantly higher than those of the control group. Although there was no statistical difference in absorbance between the 500 μg/ml and 1000 μg/ml groups, the absorbance value was higher in the 500 μg/ml group. Therefore, this concentration was selected for further exploration of material safety through live/dead staining experiments. Fig. 4B shows typical live/dead staining images of PEEK, DSPEEK, and ZnMet@PEEK (500 μg/ml) samples. Significantly, no dead cells were observed in any group at each time point, and the living cell increased markedly over time in all groups. Compared to PEEK and DSPEEK, the ZnMet@PEEK samples exhibited a more pronounced increase in cell proliferation at all time points. The results shown in Fig. 4 indicate that ZnMet@PEEK at 500 μg/ml exhibits enhanced cell viability. Therefore, this specific ZnMet@PEEK group was uniformly adopted for subsequent experiments of bioactivities assessment.

**Fig. 5:**
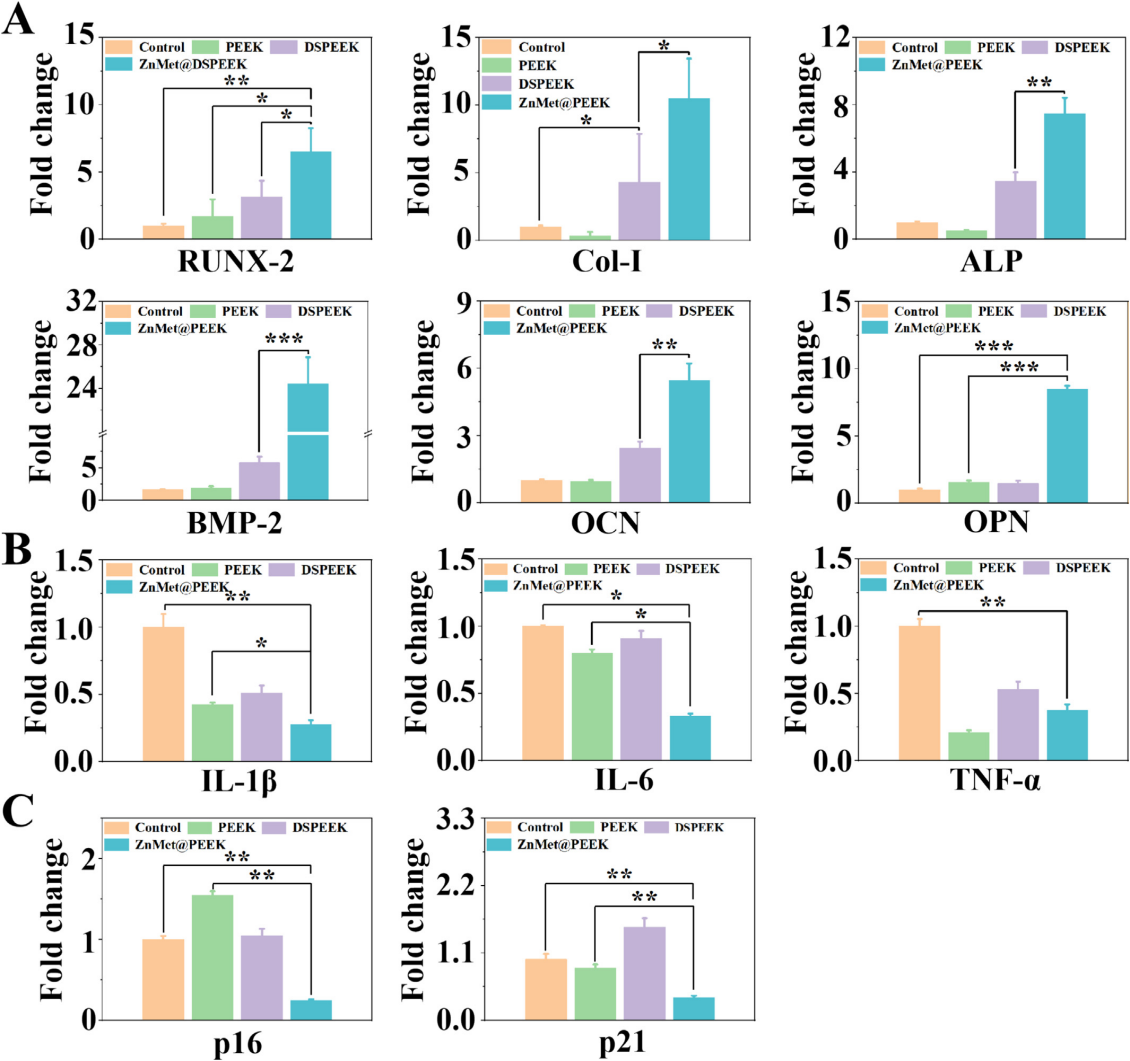
ZnMet@PEEK significantly upregulates osteogenesis-related genes, while downregulate ainflammation-related genes and aging-related genes *in vitro*. It is found by RT-qPCR that ZnMet@PEEK promotes the expression of osteogenesis-related genes (RUNX-2, Col-1, ALP, BMP-2, OCN and OPN) (A), while suppressing the expression of inflammation-related genes (IL-1β, IL-6 and TNF-α) (B) and senescence-related genes (p16 and p21) (C).

The regulatory effects of various PEEK materials on osteogenesis and anti-inflammatory related genes in MC3T3-E1 cells was investigated, as shown in Fig. 5. Here, conventional culture dishes were employed as the control group for comparative analysis. Regarding the expression of osteogenesis-related genes (RUNX-2, Col-1, ALP, BMP-2, OCN and OPN) (Fig. 5A), compared to the control group, the modified DSPEEK group demonstrated even higher osteogenesis-related genes expression than the PEEK group, further enhancing the potential for osteogenic differentiation. It is worth mentioning that the ZnMet@PEEK group exhibited the most significant increase in osteogenesis-related genes (RUNX-2, Col-1, ALP, BMP-2, OCN and OPN) expression, suggesting that ZnMet@PEEK may possess the optimal ability to promote osteogenic differentiation. Remarkably, the ZnMet@PEEK group demonstrated the most prominent upregulation of BMP-2, Col-1 and OPN, significantly higher than the DSPEEK group, further confirming its advantage in osteogenic differentiation. Regarding the inflammation-related genes IL-1β, IL-6 and TNF-α (Fig. 5B), we also conducted detailed examinations. The results revealed that, in terms of IL-1β, IL-6 and TNF-α expression, all PEEK groups exhibited significantly lower expression levels compared to the control group. Notably, the ZnMet@PEEK group demonstrated the most significant downregulation, suggesting that ZnMet@PEEK may possess the strongest anti-inflammatory effect. Although the DSPEEK group had slightly higher expression than the PEEK group, it was still lower than the control group. It is noteworthy that the ZnMet@PEEK group exhibited the lowest expression of inflammation-related genes, further confirming its significant anti-inflammatory effect. Fig. 5C demonstrated that the expression of senescence-related genes p16 and p21 in the ZnMet@PEEK group was also significantly lower than that in the control group, suggesting that the preparation of ZnMet@PEEK may possess an inhibitory effect on the expression of senescence genes.

**Fig. 6:**
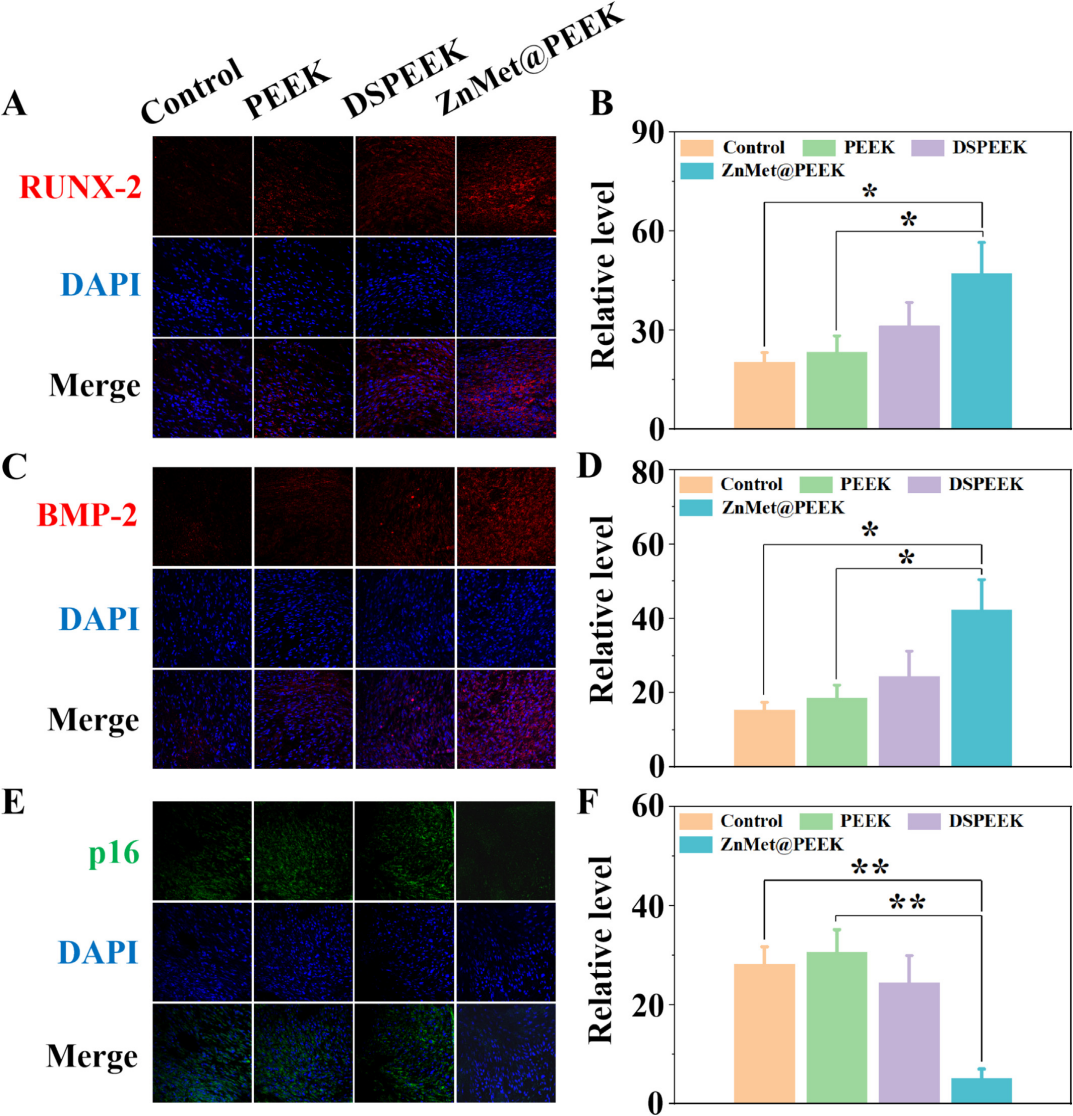
ZnMet@PEEK significantly promotes osteogenesis and inhibits cellular senescence *in vitro*. (A, C, E) It was found by immunofluorescence staining that ZnMet@PEEK significantly promotes the expression of runt-related transcription factor 2 (RUNX-2) (A), bone morphogenetic protein-2 (BMP-2) (C) and aging-related proteins (p16) (E) in MC3T3-E1 cells *in vitro*. (B, D, F) Quantitative analysis of the expression of the RUNX-2 (B), BMP-2 (D) and p16 (F).

To further investigate the osteogenesis effect of ZnMet@PEEK *in vitro*, we performed immunofluorescence staining of osteogenesis-related proteins in induced differentiated MC3T3-E1 cells from each group. Runt-related transcription factor 2 (RUNX-2) and bone morphogenetic protein-2 (BMP-2) are key factors regulating bone formation [[36], [37], [38]]. Fig. 6A, C showed that immunofluorescent staining revealed markedly enhanced expression of RUNX-2 and BMP-2 in the ZnMet@PEEK group, as evidenced by statistical analysis of mean fluorescence intensity (Fig. 6B, D). This robust osteogenic response demonstrates the synergistic effect of Zn^2+^ and Met. The synergistic activation to RUNX-2 and BMP-2 by Zn and Met is pivotal for overcoming age-related osteogenic impairment. Zn orchestrates Wnt/β-catenin signaling and epigenetic remodeling, while Met fine-tunes energy metabolism via AMPK/mTOR [[39], [40], [41]]. This dual-pathway strategy bypasses the “Wnt inhibition barrier” in aged bone microenvironment. Notably, RUNX-2 and BMP-2 upregulation directly correlates with enhanced mineralization, establishing a causal link between molecular targeting and functional output [38,42]. Fig. 6E demonstrated that the expression of senescence-related protein p16 in the ZnMet@PEEK group was also significantly lower than that in the control group, suggesting that the preparation of ZnMet@PEEK may possess an inhibitory effect on the expression of senescence protein, as evidenced by statistical analysis of mean fluorescence intensity (Fig. 6F). Some studies have reported that the synergistic targeting of p16-mediated senescence by Zn and metformin is pivotal for reversing age-related osteogenic decline, where p16 acts as a molecular brake that is differentially modulated by these two agents [43,44].

**Fig. 7:**
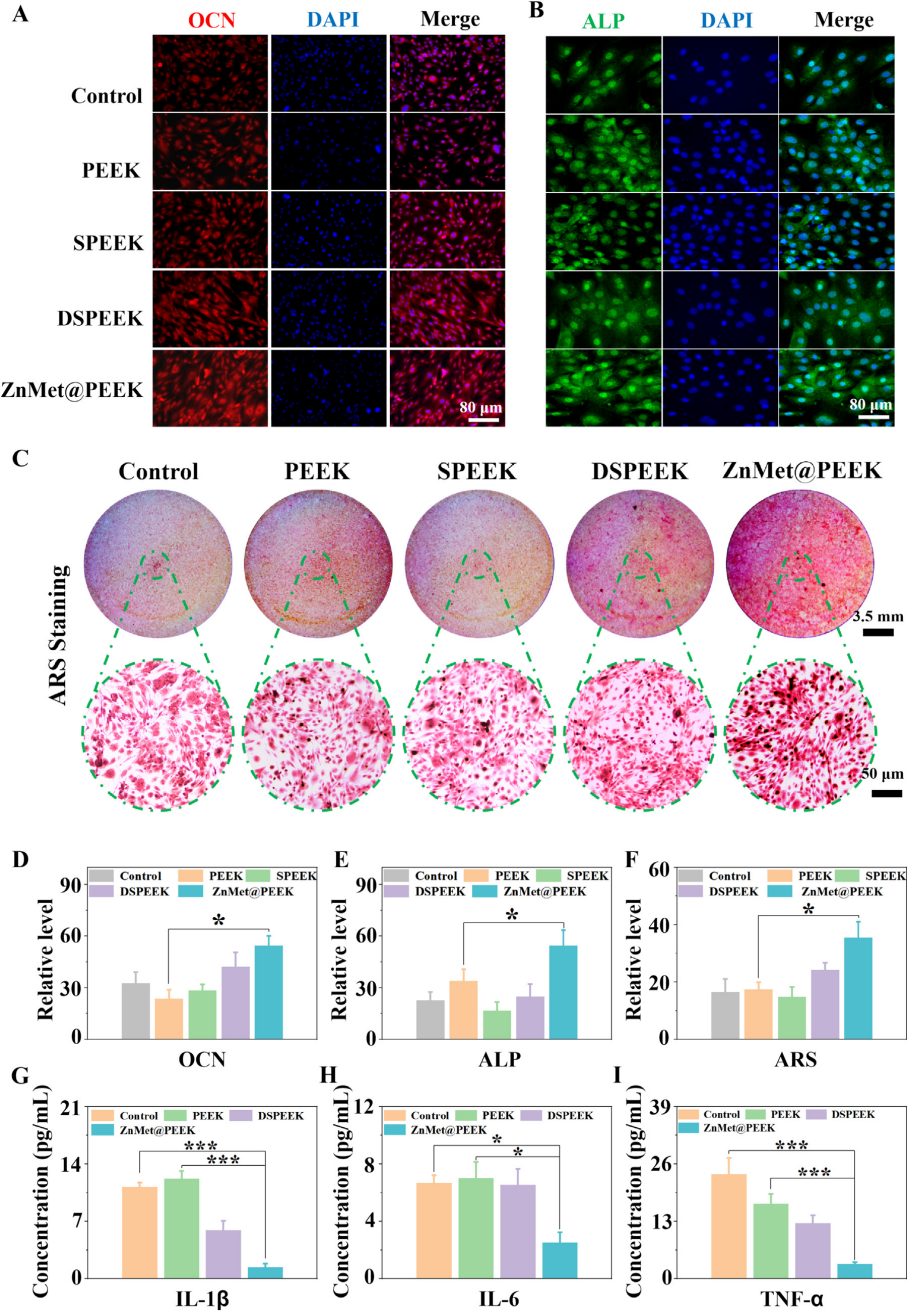
ZnMet@PEEK significantly promotes osteogenesis and anti-inflammation *in vitro*. (A-B) It was found by immunofluorescence staining that ZnMet@PEEK significantly promotes the expression of osteocalcin (OCN) (A) and alkaline phosphatase (ALP) (B) in MC3T3-E1 cells *in vitro*. (C) It was found by alizarin red staining (ARS) that ZnMet@PEEK significantly promotes bone mineralization nodules (BMN) deposition *in vitro*. (D–F) Quantitative analysis of the expression of the OCN (D), ALP (E) and BMN (F). (G–I) It was found by enzyme-linked immunosorbent assay (ELISA) that ZnMet@PEEK inhibits the expression of inflammatory factors[IL-1β (G), IL-6 (H), TNF-α (I)] *in vitro*.

The osteogenic differentiation and bone mineralization capabilities of MC3T3-E1 cells on various material surfaces were also evaluated, with the control being the standard cell culture dish surface. Fig. 7A-B presents the immunofluorescence staining images of OCN and ALP after 5 days of culturing on different sample surfaces. Notably, the ZnMet@PEEK group exhibited higher OCN and ALP expression compared to the other groups. This observation was corroborated by the quantitative analysis based on the staining images shown in Fig. 7D-E, where the ZnMet@PEEK group demonstrated significantly higher fluorescence intensity than the rest of the groups. Interestingly, the PEEK group also showed a marked increase in OCN and ALP fluorescence expression compared to the control, indicating its potential to enhance osteogenic differentiation. Furthermore, Fig. 7C displays the Alizarin red staining (ARS) images, which revealed more pronounced calcified nodules in the ZnMet@PEEK group, suggesting its superior ability to promote bone mineralization nodules (BMN). Quantitative analysis revealed the ZnMet@PEEK group exhibited highest BMN expression (Fig. 7F), implying that ZnMet@PEEK could effectively promote osteogenesis. As shown in Fig. 7G–I, the result of ELISA reveals that the levels of IL-1β, IL-6, and TNF-α decreased in ZnMet@PEEK group in comparison with the other groups, suggesting that the treatment of ZnMet@PEEK markedly promoted the production of the inflammation-related factors in MC3T3-E1 cells. The staining results, coupled with the gene expression data, highlight the potential of ZnMet@PEEK as a promising material for dental implants, given its ability to enhance osteogenic differentiation and bone mineralization while effectively reducing inflammation-related gene expression.

### *In vivo* assessment of ZnMet@PEEK for Orthopedic implants

**Fig. 8:**
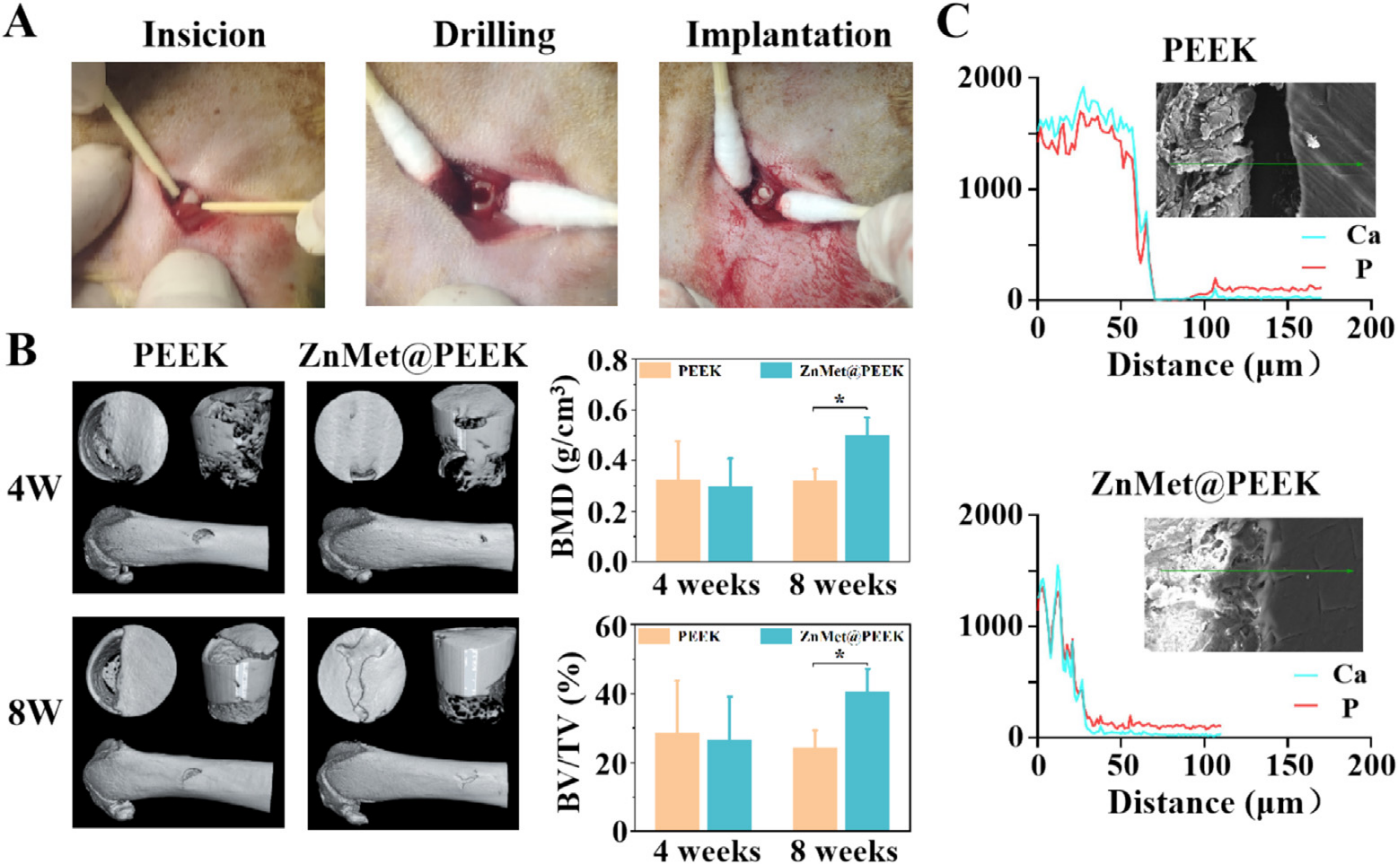
ZnMet@PEEK enhances osseointegration *in vivo* by improving bone regeneration and mineral deposition at the implant–bone interface. (A) Representative images show the surgical procedure for establishing a femoral defect model, including incision, drilling, and implantation steps. (B) Micro-CT reconstructions at 4 and 8 weeks post-implantation reveal greater bone formation around ZnMet@PEEK implants compared to PEEK; quantitative analysis shows significantly higher BMD and BV/TV in the ZnMet@PEEK group, particularly at 8 weeks (n = 3). (C) EDS line scan analysis across the implant–bone interface demonstrates deeper and more gradual elemental penetration of calcium and phosphorus in ZnMet@PEEK, indicating superior mineral integration versus the sharp element drop observed in the PEEK group.

To evaluate the osseointegration capabilities of the dental implant materials, we utilized a femoral defect model in accordance with established standard protocols from previous studies. Fig. 8 presents the preliminary results of the animal experiments assessing bone integration for PEEK and ZnMet@PEEK. The process of constructing the animal model is outlined in Fig. 8A. At 4 and 8 weeks post-implantation, femoral tissue samples, including the implants, were collected. Gross examination of the tissues indicated no evidence of infection in any samples, and ZnMet@PEEK exhibited improved bone integration compared to PEEK. To validate these visual observations, micro-CT scanning analysis and EDS line scans across the implant-bone interface were performed. The micro-CT images presented in Fig. 8B confirm the visual assessment, showing a higher amount of new bone formation around ZnMet@PEEK compared to PEEK, particularly evident at 8 weeks post-implantation. This observation is further supported by quantitative analyses based on CT scan data, which indicate significantly higher BMD and BV/TV values around ZnMet@PEEK implants at 8 weeks compared to PEEK implants. Furthermore, EDS line scan results presented in Fig. 8C reveal distinct differences in the distribution of calcium and phosphorus elements across the implant-bone interface between the two groups. In the PEEK group, a sharp decrease in calcium and phosphorus concentrations is observed as the scan crosses the interface, indicating limited bone integration. In contrast, the ZnMet@PEEK group shows a more gradual decline in these element concentrations, suggesting better integration between the modified implant and bone tissue.

**Fig. 9:**
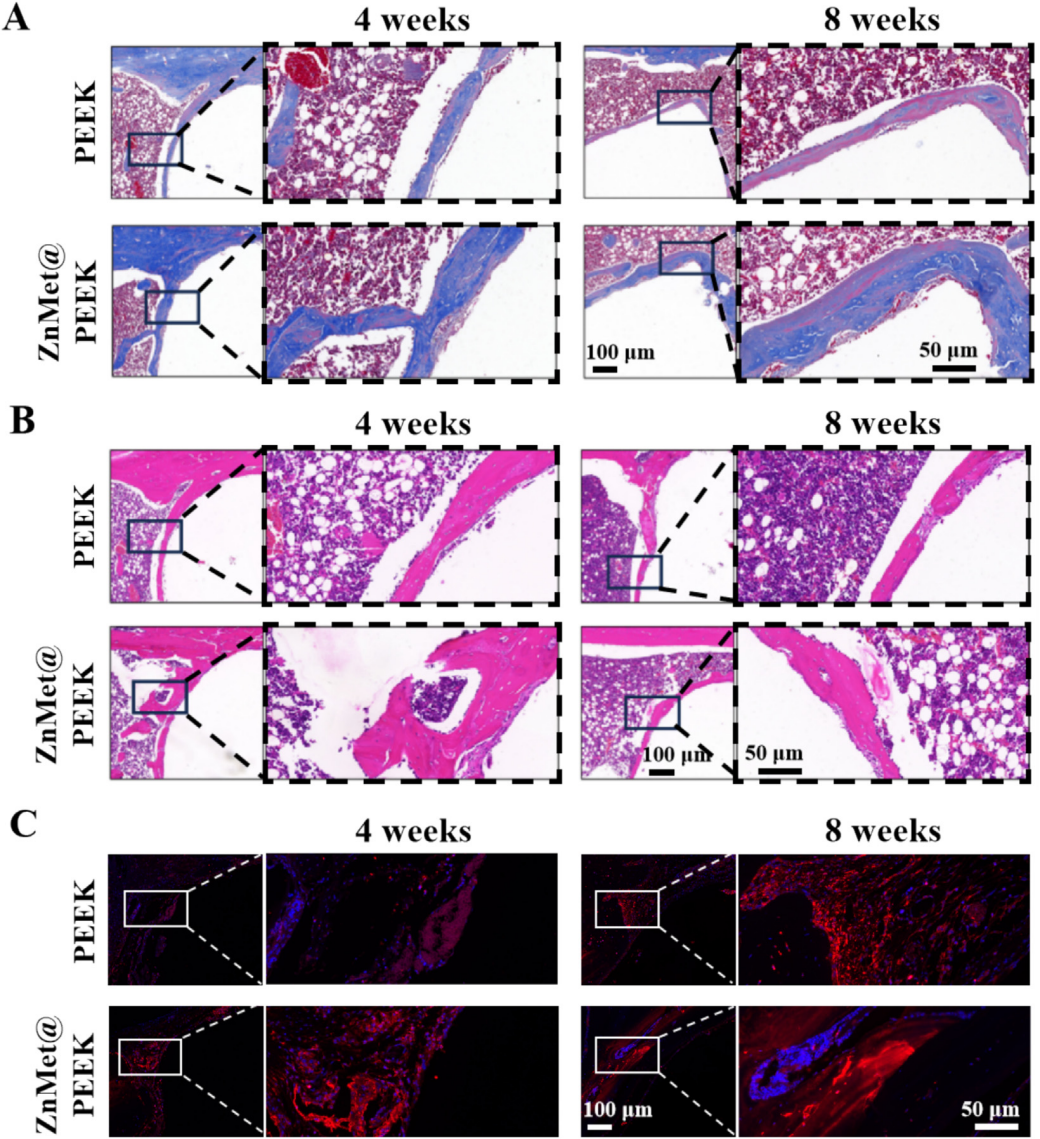
Histological staining reveals enhanced collagen organization and cellular activity around ZnMet@PEEK implants. (A) Masson’s trichrome staining (top panels) shows more abundant and uniformly aligned collagen fibers (blue) at the implant–bone interface in the ZnMet@PEEK group compared to the PEEK group at both 4 and 8 weeks, indicating improved extracellular matrix remodeling. (B) H&E staining (bottom panels) demonstrates greater cellular density and new bone formation adjacent to ZnMet@PEEK implants, especially at 8 weeks, suggesting accelerated bone regeneration and superior tissue integration. (C) Immunofluorescence staining (bottom panels) demonstrates more RUNX-2 expression to ZnMet@PEEK implants, especially at 8 weeks, suggesting accelerated bone formation and superior tissue integration. All images are representative of three animals per group (n = 3); boxed regions are shown at higher magnification.

The histological analysis presented in Fig. 9 provides further evidence of better osteointegration capabilities of ZnMet@PEEK, as compared to the PEEK group. Masson staining (Fig. 9A) reveals a notably higher distribution of collagen fibers for ZnMet@PEEK samples. These collagen fibers are not only more abundant but also exhibit a uniform arrangement, indicative of improved bone formation and matrix organization. This observation suggests that the ZnMet@PEEK composite fosters a more conducive environment for collagen deposition, a crucial step in bone regeneration. Consistent with the Masson's trichrome findings, HE staining (Fig. 9B) further corroborates the superior osteogenic response elicited by ZnMet@PEEK. The HE stained images reveal more pronounced cortical bone formation in the vicinity of the osseointegration interface in the ZnMet@PEEK group compared to the PEEK group. To further investigate the osteogenic potential of ZnMet@PEEK *in vivo*, we also performed immunofluorescence staining for the osteogenesis-related protein RUNX-2 in each experimental group. As shown in Fig. 9C, RUNX-2 expression was strongest in the ZnMet@PEEK group, especially at 8 weeks, indicating that ZnMet@PEEK promotes bone regeneration by accelerating bone metabolism and osteoblast differentiation, and is superior tissue integration..

**Fig. 10:**
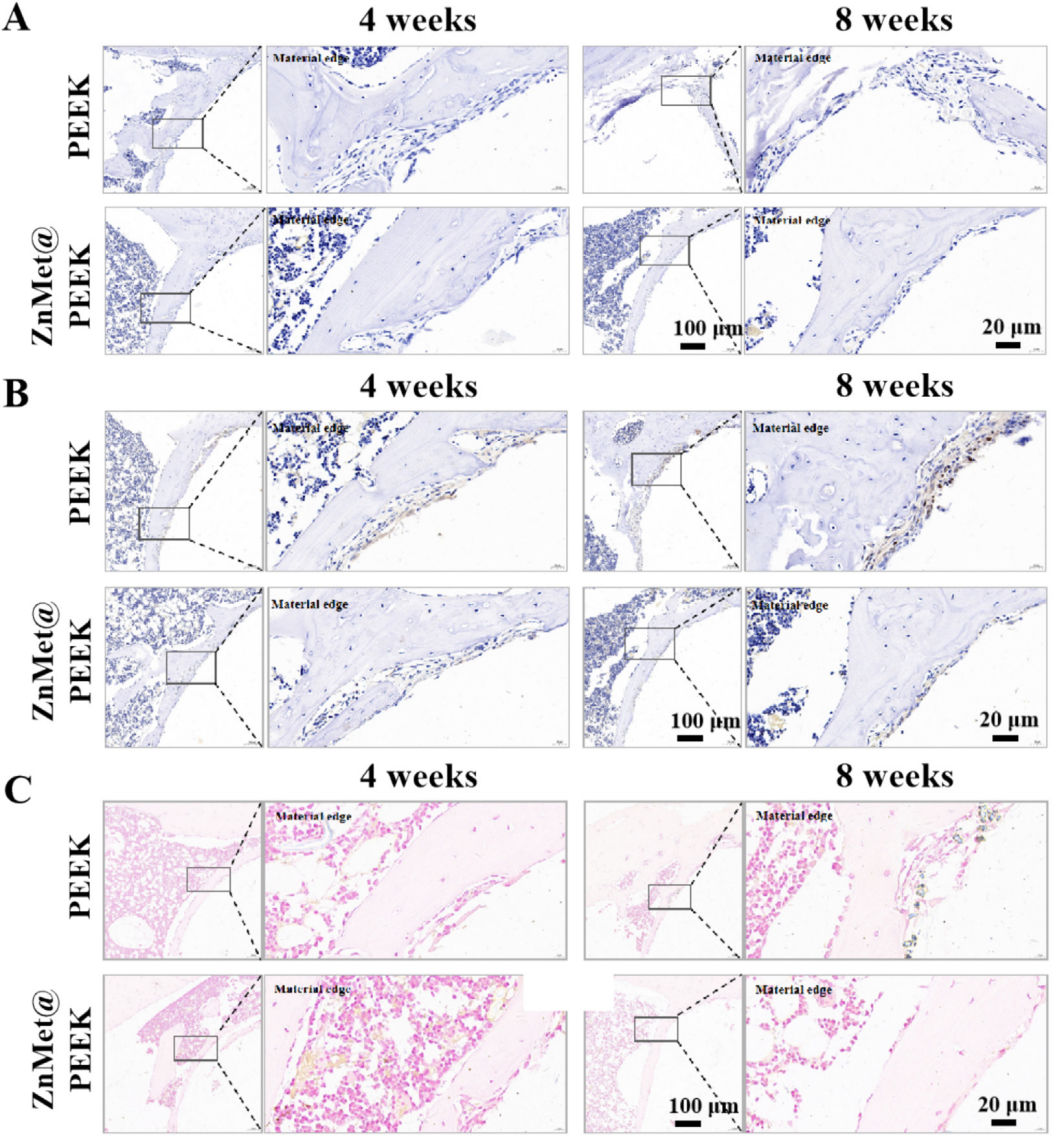
ZnMet@PEEK reduces cellular senescence at the implant interface in aged rats. Immunohistochemical staining of senescence-associated markers was performed to evaluate local tissue aging. (A) P53 expression shows no notable difference between ZnMet@PEEK and PEEK at either 4 or 8 weeks, suggesting minimal impact on early senescence stress signaling. (B) P21 expression is markedly reduced in the ZnMet@PEEK group at both time points, indicating effective inhibition of senescence-associated cell cycle arrest at the material–bone interface. (C) β-galactosidase staining (combined with H&E) reveals fewer β-gal^+^ senescent cells in ZnMet@PEEK-treated tissues, particularly near the implant edge, compared to PEEK, especially at 8 weeks. All results are representative of three independent animals per group (n = 3); boxed areas show magnified views of material-tissue interfaces.

To investigate the potential ameliorative effects of ZnMet@PEEK on cellular senescence that impacts tissue regeneration efficiency prominently in elderly individuals due to their inherently reduced regenerative capacity, we conducted immunohistochemical staining for P53, P21, and combined hematoxylin and eosin (HE) with β-gal staining on tissue sections. The results are presented in Fig. 10. Fig. 10A illustrates the P53 staining outcomes. Notably, there were no significant differences in P53 expression between the PEEK group and the ZnMet@PEEK group at both the 4th and 8th weeks post-operation. This suggests that ZnMet@PEEK does not significantly alter P53 expression levels, a key regulator of cellular senescence and stress response. Fig. 10B displays the P21 staining results. Interestingly, a reduced expression of P21 was observed in the ZnMet@PEEK group compared to the PEEK group at both the 4th and 8th weeks. P21, a cyclin-dependent kinase inhibitor, plays a crucial role in cell cycle arrest and senescence [45,46]. Its decreased expression in the ZnMet@PEEK group indicates a potential attenuation of cellular senescence processes. Lastly, Fig. 10C presents the combined HE and β-gal staining results. At the 8th week, a discernible difference emerged, with the ZnMet@PEEK group exhibiting lesser β-gal expression. β-gal, a marker of cellular senescence, was detected in blue-stained cells within normal bone tissue in the PEEK group, suggesting the presence of senescent cells potentially associated with aging. Similarly, blue-stained cells were observed at the interface between the PEEK material and bone tissue, indicating the presence of senescent cells in this region. However, in the ZnMet@PEEK group, while blue-stained cells were still present within the bone tissue, they were noticeably absent at the interface between the material and bone, implying a reduction in senescent cells in this critical area. These findings suggest that ZnMet@PEEK may exert a positive effect on mitigating cellular senescence in the vicinity of implanted materials, particularly at the material-bone interface, which could ultimately enhance tissue regeneration efficiency.

**Fig. 11:**
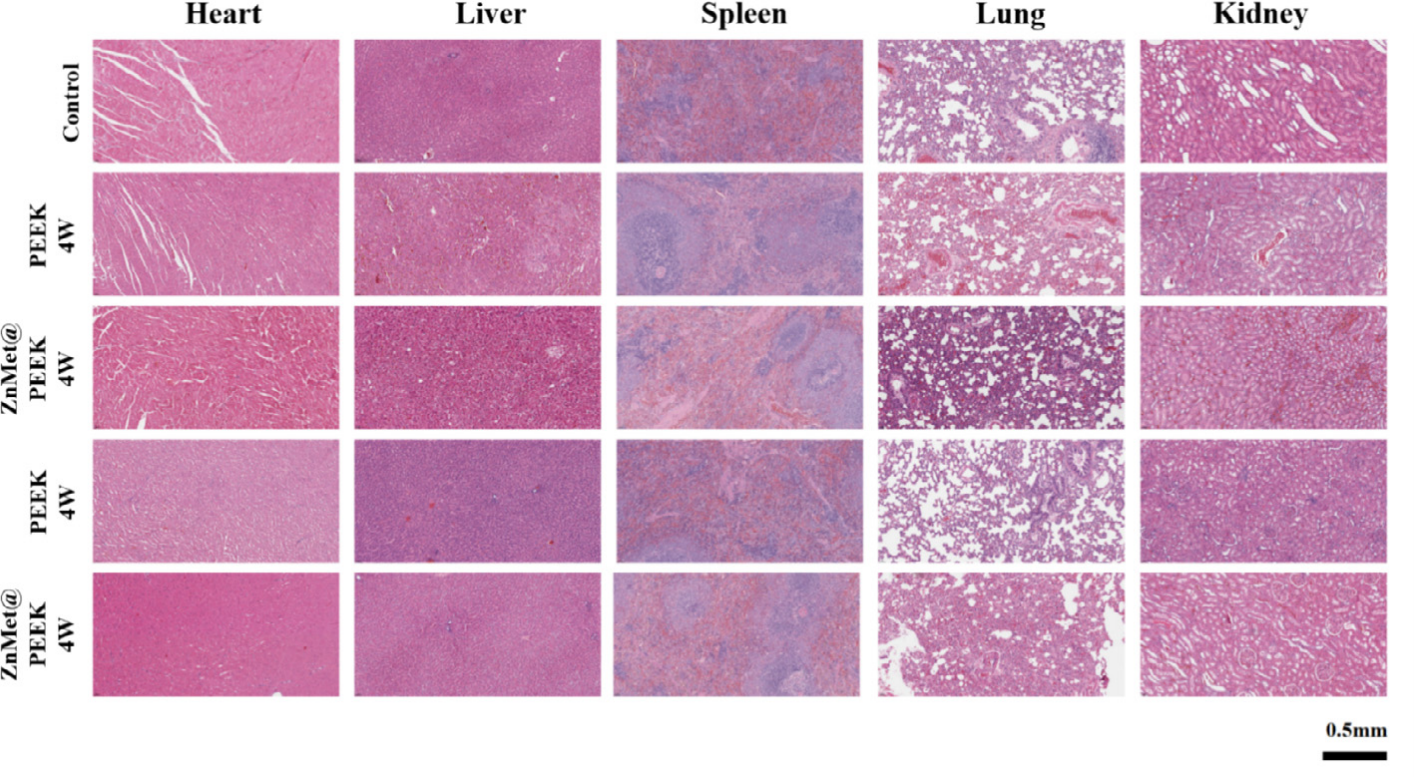
ZnMet@PEEK demonstrates no observable systemic toxicity in major organs. Representative hematoxylin–eosin (H&E) stained sections of heart, liver, spleen, lung, and kidney harvested from rats 4 and 8 weeks after implantation of PEEK or ZnMet@PEEK. Tissue morphology in all groups, including the control, shows no evidence of inflammation, necrosis, cellular degeneration, or pathological changes. ZnMet@PEEK-treated rats exhibit comparable histoarchitecture to control and PEEK groups, confirming good *in vivo* biocompatibility and systemic safety. Scale bar = 500 μm. Representative images from three rats per group (n = 3).

Fig. 11 presents the H&E staining outcomes of vital organs following 4 and 8 weeks after surgery, with implantation of PEEK and ZnMet@PEEK. Notably, no discernible tissue damage was observed in the critical organs across all groups, encompassing the heart, liver, spleen, lung, and kidney. This observation suggests that the ZnMet@PEEK sample we developed should be clinical safety *in vivo*.

## Discussion

To address the multifaceted challenges of dental implant therapy in the elderly, a functionalized PEEK derivative, ZnMet@PEEK, has been developed by incorporating Zn and Met onto the PEEK surface using a dopamine-assisted physical adhesion method. This surface modification strategy seeks to enhance PEEK's bioactivity by leveraging the synergistic effects of Zn and Met. A series of physicochemical characterizations have been conducted to confirm the successful preparation of ZnMet@PEEK. The dopamine-assisted functionalization strategy employed here offers distinct advantages over existing PEEK surface modifications. 1. Preservation of bioactivity: unlike covalent grafting (e.g., plasma treatment or silanization), which may denature thermolabile agents like metformin, dopamine’s physical adhesion via π-π stacking and hydrogen bonding maintains ZnMet’s structural integrity and dual functionality (validated by FTIR/UV–Vis in Fig. 3A–C) [3,47]. 2. Enhanced payload capacity: sulfonation-induced micropores (0.5–5 μm, Fig. 2D) provide a high-surface-area scaffold for dopamine anchoring. This enables >500 μg/mL ZnMet loading (Fig. 4A), surpassing the limited drug-carrying capacity of thin chemical coatings (e.g., layer-by-layer assembly) [3]. 3. Controlled Release Kinetics: the biphasic release profile (rapid initial phase for anti-inflammatory action, plateau for sustained osteogenesis, Fig. 3F–G) arises from ZnMet’s distribution within porous structures rather than surface-only binding. This contrasts with electrodeposition or spray coating, where burst release dominates [48]. Clinical Scalability: Dopamine immersion is solvent-free, ambient-temperature, and scalable—advantages over complex techniques like CVD or PVD, which require high-energy inputs and lack compatibility with bioactive molecules[49,50].

The enhanced bone-implant integration observed in aged rats (Fig. 8, Fig. 9, Fig. 10) stems from ZnMet@PEEK’s concurrent targeting of the inflammatory-senescence-osteogenic triad that characterizes elderly bone regeneration. Zinc ions (Zn2^+^) orchestrate a dual-pathway response: (1) NF-κB suppression mitigates TNF-α/IL-6-driven bone resorption and inhibits p16 transcription via ROS scavenging [51,52]; (2) Wnt/β-catenin activation (potentiated via miR-665/SOST) rescues age-diminished osteogenesis, evidenced by RUNX-2/BMP-2 upregulation (Fig. 5, Fig. 6) [53,54]. Metformin complements this by (1) augmenting BMP-2 signaling through AMPK-mediated clearance of antagonists (e.g., Noggin), (2) suppressing senescence via mTOR inhibition and SIRT1-dependent epigenetic silencing of p16/p21, and (3) attenuating SASP-mediated matrix degradation [27,[55], [56], [57], [58], [59]]. This combinatorial strategy disrupts the vicious cycle linking inflammation, senescence, and Wnt inhibition in aged microenvironments—a critical advance over single-factor modifications (e.g., hydroxyapatite coatings). The dopamine-assisted functionalization is pivotal to this synergy. Unlike covalent grafting (e.g., silanization) that risks denaturing metformin, physical adsorption preserves bioactivity while leveraging sulfonated PEEK’s microporosity (>500 μg/mL payload capacity) for controlled biphasic release: rapid Zn^2+^/Met elution counters acute inflammation, followed by sustained osteogenic modulation [47]. This 3D storage strategy surpasses monolayer coatings (e.g., electrodeposition) in drug-loading efficiency and outperforms layer-by-layer assembly in scalability for clinical translation.

ZnMet@PEEK’s success in aged models highlights its potential to bridge the “osteogenic gap” in elderly implantology. The material’s rapid wettability (Fig. 3D-E) and porosity facilitate early cell adhesion and mass transport, accelerating osseointegration—critical for patients with compromised healing [60,61]. However, long-term senescence modulation warrants further study; while P21/β-gal were reduced (Fig. 10), P53 persistence suggests additional senescence pathways may require targeting. Future work should explore in situ ZnMet replenishment via injectable hydrogels or compare dopamine adhesion with polydopamine polymerization for stability. Nevertheless, this study establishes dopamine-assisted ZnMet loading as a versatile platform for multi-agent delivery on PEEK, adaptable to other implants facing age-related challenges.

## Conclusions

In this study, we developed ZnMet@PEEK, a functionalized PEEK derivative with anti-inflammatory, osteogenic, and anti-senescence properties, tailored for elderly dental implant patients. Physicochemical characterization confirmed the successful synthesis of ZnMet@PEEK, and *in vitro* experiments further demonstrated its remarkable multifunctional bioactivity, particularly its osteogenic, and anti-inflammatory properties. Specifically, ZnMet@PEEK promotes osteoblast differentiation and bone mineralization while effectively attenuating inflammatory responses and mitigating cellular senescence. These findings were further validated through therapeutic evaluation using a rat femoral defect model, which exhibited enhanced bone formation at the implant-bone interface, increased bone mineral density, and reduced cellular senescence. Importantly, ZnMet@PEEK demonstrated excellent biocompatibility, with no significant tissue damage to critical organs, thereby ensuring its clinical safety. Therefore, we believe that ZnMet@PEEK should be a promising dental implant material for the elderly, offering a novel solution to the multifaceted challenges of osseointegration.

## Consent for publication

The authors confirm that written informed consent for publication of the study data and any potentially identifiable information or images was obtained from all participants involved in this study. In cases where participants are not legally competent to provide consent, consent was obtained from a parent, legal guardian, or legally authorized representative. All procedures were conducted in accordance with institutional and national ethical standards.

## Availability of data and material

The datasets used and/or analyzed during the current study are available from the corresponding author on reasonable request.

## Compliance with Ethics Requirements

All procedures performed in this study involving animals were conducted in accordance with the ethical standards of the institutional and/or national research committee and with the 1964 Helsinki Declaration and its later amendments or comparable ethical standards. Informed consent was obtained from all individual participants included in the study.

Ethics approval was obtained from the Animal Experiment Ethics Committee of Southern University of Science and Technology, with approval number 20220829.

## Funding

We acknowledge the support received from Guangxi Science and Technology Plan Project (Guike AA25069007), the Guangdong provincial key laboratory of advanced biomaterials (2022B1212010003), the Shenzhen Science and Technology Innovation Committee (KCXFZ20240903093917023, KCXFZ20211020174805009), and the talent research project of Shenzhen Natural Science Foundation (Grant No. RCBS20221008093233049).

## Declaration of competing interest

The authors declare that they have no known competing financial interests or personal relationships that could have appeared to influence the work reported in this paper.
