# Supplementary material for: Zinc and metformin co-functionalized polyetheretherketone: A novel dental implant material tailored for the elderly
Source: J Adv Res. 2025 Aug 27;84:477–91. doi: 10.1016/j.jare.2025.08.056 (PMC13227235; doi:10.1016/j.jare.2025.08.056)
Supplement: Supplementary Data 1 [file mmc1.docx]

Supporting Information

**Zinc and Metformin Co-Functionalized Polyetheretherketone: A Novel Dental Implant Material Tailored for the Elderly**

Table of Content

**Table S1.** Biochemical Reagents

**Table S2.** Experimental Equipment

**Table S3.** Comparative performance of ZnMet@PEEK and representative modified PEEK materials in terms of synthesis complexity, biocompatibility, mechanical strength, anti-inflammatory potential, anti-senescence efficacy, and osteointegration.

**Table S1.** Biochemical Reagents

| **Reagent Name** | **Catalog Number** | **Manufacturer** |
| --- | --- | --- |
| **DMEM** | G4524-500ML | Saviore, China |
| α**-MEM** | G4555-500ML | Saviore, China |
| **CCK-8** | K1018 | APEXBIO, China |
| **DEPC-treated Water** | B501005-0500 | Sangon Biotech, China |
| **AG Evo M-MLV Reverse Transcriptase** | AG11706 | Accurate Biotechnology, China |
| **AG SYBR Green Reagent** | AG11701 | Accurate Biotechnology, China |
| **Trizol RNA Extraction Reagent** | R0016 | Beyotime, China |
| **Chloroform** | C805077 | Macklin, China |
| **Isopropanol** | I811932 | Macklin, China |
| **Absolute Ethanol** | E809056 | Macklin, China |

**Table S2.** Experimental Equipment

| Name | Catalog Number | Manufacturer |
| --- | --- | --- |
| Scanning Electron Microscope (SEM) | JSM-7200F | JEOL, Japan |
| Scanning Electron Microscope (SEM) | Nova NanoSem450 | FEI, USA |
| ATR-FTIR Spectrometer | VERTEX 70v | Bruker, Germany |
| UV–Visible Spectrophotometer | UV-2600 | SHIMADZU, Japan |
| ICP-MS | 7900 ICP-MS | Agilent, USA |
| Contact Angle Measuring System | VCA OPTIMA | AST, USA |
| -80 ℃ Freezer | BCD-285 | Haier, China |
| Ultrapure Water System | GenPure UF | Thermo Scientific, USA |
| Magnetic Stirrer | WH-610D | IKA, Germany |
| X-ray Diffractometer | Empyrean | PANalytical, Germany |
| Analytical Balance | BSA124S | Sartorius, Germany |
| Clean Bench | 1300-A2 | Thermo, USA |
| CO₂ Incubator | Hera Cell 150 | Thermo, USA |
| Constant Temp Shaker | HY-58 | Qianyan Tech, China |
| Microplate Reader | SPARK | Tecan, Switzerland |
| Microvolume Spectrophotometer | NanoDrop | Thermo, USA |
| Inverted Microscope | Eclipse Ts2 | Nikon, Japan |
| Autoclave | GR60DA | Zealway, USA |
| Ultrasonic Cleaner | SB-100DT | Xinzhi Instruments, China |
| Drying Oven | DHG-9075A | Yiheng Scientific, China |
| RT-qPCR | Biosystem ABI | Thermo Scientific, USA |
| PCR Machine | T100 | Bio-Rad, Germany |
| CO₂ Incubator | Il6 | Thermo Scientific, USA |
| Inverted Fluorescence Microscope | DMI8 | Leica, Germany |
| High-speed Centrifuge | D3024R | Thermo Scientific, USA |
| Biosafety Cabinet | ESCO | ESCO, Singapore |
| 4 ℃ Refrigerator | YCD-EL259A | Meiling, China |
| Clean Bench | Esco | ESCO, Singapore |
| Pipette Set | 2.5/10/20/100/200/1000μL | Gilson/Eppendorf, Germany |
| Syringe | Jie Rui | Weigao, China |
| Two-photon Confocal Microscope | LSM 980 | Carl Zeiss, Germany |
| Vortex Mixer | QL-901 | Qilinbeier, China |
| Microplate Reader | iMark | Bio-Rad, Germany |
| Microvolume Spectrophotometer | Nanodrop2000 | Thermo Scientific, USA |
| PVDF Membrane | Bio-rad | Bio-rad, Germany |
| Sterile Filter Membrane | 0.22 µm | Millipore, Germany |

**Table S3.** Comparative performance of ZnMet@PEEK and representative modified PEEK materials in terms of synthesis complexity, biocompatibility, mechanical strength, anti-inflammatory potential, anti-senescence efficacy, and osteointegration.

| Platform Name | Synthesis Complexity (Y) | Biocompatibility (L) | Mechanical Strength (S) | Anti‑Inflammatory (A) | Anti‑Senescence (E) | Osteointegration (O) |
| --- | --- | --- | --- | --- | --- | --- |
| ZnMet@PEEK | Multi‑step: Dopamine-assisted adhesion → Zn + Met loading → freeze-drying | Excellent: Zn & Metformin enhance osteogenesis and anti-inflammation via AMPK | Moderate: PEEK modulus ~3–4 GPa | Excellent: Zn downregulates IL‑1β, IL‑6, TNF‑α; Met reduces inflammation | Excellent: Metformin activates AMPK, reduces P21 and β-gal | Excellent: Increased bone formation and mineral density in aged rats |
| PEEK/HA | Multi‑step: HA coating or blending | Excellent: HA is osteoconductive and biocompatible | High: Modulus ~7–10 GPa with HA content | Moderate: HA provides mild anti-inflammatory effects | Poor: No evidence of anti-senescence activity | Good: HA significantly improves osseointegration |
| CFR‑PEEK | Multi‑step: Carbon-fiber reinforcement | Good: Bioinert—minimal tissue response | Very High: Strength ~100–150 MPa, modulus ~20–150 GPa | Poor: No bioactive anti-inflammatory properties | Poor: No anti-senescence benefit | Poor: Bioinert—limited bone bonding |
| PEEK/TiO₂ | Multi‑step: TiO₂ incorporation (e.g., PDA‑mediated or melt blending) | Good: Improved cell adhesion | High: Modulus ~4–5.5 GPa, improved hardness | Moderate: TiO₂ mildly reduces ROS/inflammation | Poor: No evidence of anti-senescence activity | Moderate: TiO₂ enhances early osteogenesis |
